# The Family Practitioner's role in the integrated continuum of cancer care

**DOI:** 10.4102/phcfm.v7i1.821

**Published:** 2015-02-11

**Authors:** Martin de Villiers

**Affiliations:** 1 Independent Clinical Oncology Network, South Africa

According to the World Health Organization (WHO), cancer and non-communicable diseases have become a global epidemic.^1^ The WHO estimates that 60% of the people developing cancer for the first time live in Africa, Asia and Central or South America and, furthermore, that 30% of cancers can be prevented.

The age-standardised incidence of cancer in South Africa is estimated to be 170/100 000 women and 254/100 000 men.^2^ Although the incidence of cancer varies widely in different parts of the world and is generally reported as being significantly higher in high income countries – in many cases more than double that of low- and middle-income countries – it is reported that the incidence of cancer in sub-Saharan Africa is rising at an alarming rate. The proportion of people developing cancer for the first time from low- and middle-income countries will rise to 70% by 2030.^3^

It is within this context of the growing cancer burden in sub-Saharan Africa and, specifically, resource-limited countries, that an integrated and multidisciplinary approach to cancer care needs to be implemented. In Africa, patients with cancer frequently present late and with advanced disease because of a lack of effective disease prevention, weak primary healthcare systems and difficulty in accessing specialised investigation and treatment.

Nevertheless, the family practitioner is in a unique position to implement secondary prevention and to help his or her patients with a diagnosis of cancer to navigate through the healthcare system, especially when the patient moves from one cancer phase to another, and to ensure that these patients are treated holistically. Many authors have recognised a lack of continuity in cancer care, which is sometimes because family practitioners are excluded from the care process.^4^,^5^

This growing burden of disease from cancer challenges family practitioners to take a more active role in cancer care and the mobilisation of appropriate resources in order to reduce the impact of cancer on the physical, social, psychological and financial burden of individual patients and communities.

In no other disease entity is the role of the family practitioner more important with regard to changing a disease- and doctor-centric model into a patient-centred model. Patients with cancer need to be treated as people in the context of their own families and communities, listened to, informed and respected. They need to be involved in their own care and their wishes need to be honoured during their healthcare journey. The family practitioner's role is transformed from one of authority to one of partnership, solidarity, empathy, and collaboration.^6^

Family practitioners who provide leadership at the primary care level need to build an integrated and multidisciplinary approach to cancer care. In this primary care setting, teams of doctors, nurses, social workers and community health workers need to work collaboratively with their specialist colleagues to address current short-falls on process and structural issues^7^ that impact on quality and outcomes of cancer care. This includes a community-oriented approach and technological support in order to manage identified risk factors for cancer in communities served by the primary health team.

Family practitioners need to embrace this challenge of primary care oncology as defined by Campbell et al. as ‘first-contact, continuous, comprehensive and co-ordinating care’.^8^

The main focus for the family practitioner across the continuum of cancer care includes the following:

- Raising awareness in the communities we serve on early signs and symptoms of common cancers as well as lifestyle issues, so as to contribute toward the prevention and early detection of cancer.
- Screening for common cancers in a stratified manner across healthy populations, in order to identify individuals who have disease, but do not yet have symptoms.
- The early recognition and diagnosis of suspicious cancer symptoms and signs and subsequent appropriate referral for treatment, ideally to an oncologist.
- Detection and management of distress and the psychosocial impact of the disease at diagnosis and throughout the trajectory of care.
- Continuous involvement during specialised treatment of the cancer patient to manage, amongst other things, any co-morbidities.
- Follow-up of patients after completion of authorised treatment in cooperation with the specialist or oncologist.
- Coordination of the survivorship process in patients with advanced disease with provision of support for the patient, his/her family and coordination of care when active anti-cancer treatment has been stopped or when continuing it would be futile or against the patient's wishes. Access to morphine or other opiates is a particular challenge in many African countries.
- Providing support for families and carers of cancer patients, including bereavement care.

The role of the family practitioner is key to addressing issues of fragmentation, hospital- and specialist- centeredness and over-commercialisation in healthcare, as well as to ensure an integrated, quality-driven care system with improved outcomes. Clearly, this must be done within the context of different countries, their health systems and what patients and their families can afford.

The strategic reorientation of the role of primary care supports a futuristic delivery system for cancer care that is not fragmented, but rather one that is interconnected and interdependent and will ensure that patients’ needs are met from health promotion through to end-of-life care.

In response to these challenges, the University of Stellenbosch, in collaboration with the Independent Clinical Oncology Network (ICON) in South Africa, has developed an online short-course to enable the family practitioner to develop the necessary skills and expertise (http://www.cancernet.co.za).
